# Computerized Generation of Endodontic Files by Reproducing the Flute Grinding Manufacturing Process

**DOI:** 10.3390/bioengineering11080751

**Published:** 2024-07-24

**Authors:** Victor Roda-Casanova, Antonio Pérez-González

**Affiliations:** Department of Mechanical Engineering and Construction, Universitat Jaume I, 12071 Castelló de la Plana, Spain; aperez@uji.es

**Keywords:** endodontic files, computer-aided design, computer-aided manufacturing

## Abstract

*Background*: File fracture during root canal treatment in endodontics is a major concern for clinicians. The strength of the file is strongly dependent on its geometry, material, and working conditions; finite element simulations are used to understand these failure mechanisms. One limitation of the models used for these simulations is the approximate geometric representation typically obtained by rotating and scaling a specific cross-section shape along the file length. Given the influence of file geometry on file strength, a more realistic representation based on the manufacturing method is needed. *Methods*: A computerized method was developed to generate the file geometry by simulating the flute grinding manufacturing process. This method generates the 3D geometry of the file starting from a blank and reproducing the motions of the file and grinding wheel. *Results*: The cross-section of the resulting geometry does not involve simple rotation and scaling but changes from the shank to the tip. The tilt angle of the grinding wheel affects the final geometry, thus altering the convexity of the cross-section. Several other parameters, such as the pitch and the radius of the grinding disc tip, impact the final geometry. *Conclusions*: The proposed computational method allows for the generation of endodontic file geometries that match those produced via the actual flute grinding method. This tool may help researchers and tool designers in the preparation of finite element models to assess the strength of realistic files.

## 1. Introduction

Nickel–titanium (NiTi) files, instead of stainless steel hand files, are being increasingly used for the preparation of root canals in endodontic treatment. Different manufacturers have produced files with improved geometries, materials, and manufacturing methods in an attempt to develop more efficient and durable files [1]. Some of these new instruments no longer meet the ISO 3630 standard [2], which was developed for stainless steel tools [3]. These new instruments increase the options for professionals but hinder the understanding of the suitability of different files for particular clinical cases [4].

Characteristics such as taper, cross-section shape, and helix and rake angles, as well as the separation between successive cutting blades, are critical for the clinical outcome of root canals and also affect the mechanical strength of the files. Other factors, such as the file material, thermal treatment, and the geometry of the root canal, also affect the life expectancy of the files, whose fracture during root canal preparation is one of the main concerns for clinicians. These effects have been analyzed through in vitro tests [5,6,7,8,9,10] or computer simulations [11,12,13,14]. A thorough literature review of the latter has been conducted [15].

Computer simulations using the finite element (FE) method are particularly interesting in this context, because they allow for the analysis of the effect of specific design parameters on the mechanical strength of a file, thus avoiding unwanted random changes in other parameters. FE models can be used to adequately reproduce the experimental in vitro tests of endodontic rotary files [11,12,16,17,18]. A recent review [19] showed that the finite element method is a reliable tool for evaluating the behavior of NiTi rotary instruments and reduces instrument development time and costs. Moreover, these models provide information about the stresses and strains in different file regions, thus helping to understand the reasons behind their fracture during use.

However, FE models are based on geometric models of files, which are challenging to accurately obtain due to the complex three-dimensional helical shape of a file. As such, 3D CAD or complex scanning methods must be used; however, even using these advanced techniques, the geometry of the final model will be different from that of the actual file.

Roda-Casanova [20] developed an automated procedure for simplifying the capturing of this geometry and its discretization into finite elements. However, this method is purely geometric and does not reproduce the manufacturing method followed to produce a file, which introduces geometric dissimilarities between the model and actual file. Moreover, the real geometry of a file differs from the nominal geometry [21], which is probably due to the manufacturing method. The accuracy of the geometric model of the file is important, because small differences in geometry can substantially affect crack initiation and the fatigue life of the mechanical elements that are subject to variable loads [22]. Therefore, a method is needed for generating the geometric model of a file based on the actual manufacturing method.

Different methods have been developed and used for manufacturing endodontic files. The file material influences file selection. Stainless steel files can be produced either by grinding the flutes, with subsequent grinding to obtain the taper, or by twisting the file blank with different initial sections, such as triangular or squared sections [23]. With the advent of NiTi, manufacturing methods initially shifted to grinding, because the hyperelastic behavior of this material hinders the twisting of the file to a permanent shape. However, heat treatments solved this issue, thus allowing for the use of twisting as a method for manufacturing NiTi files and paving the way for the introduction of M wires [1].

Different researchers have tried to improve the manufacturing process for endodontic files by applying machining or grinding. Several patents [24,25,26] claim to provide methods for producing files with increased flexibility and resistance to torsional breakage. These methods are based on the use of a grinding wheel that rotates, thus contacting the file blank, while being translated and rotated relative to the wheel, thus machining helical flutes. Hoppe et al. [27] patented a similar process. Some commercial machines follow this manufacturing method [28]. Taylor et al. [29] proposed an improved multipass method for grinding using a similar principle. Other machining methods were then proposed to increase the quality of the manufactured file using high-speed milling tools instead of grinding wheels [30,31]. These methods have some advantages, such as faster production speeds, the avoidance of microcracks in the file, and a lower impact on the properties of the material.

In this paper, we present a computational method that enables the automatic generation of realistic geometries of endodontic files, thus reproducing the manufacturing methods that involve the use of grinding wheels. This is a first step in more accurately capturing the geometries of endodontic files to feed FE models, with the aim of increasing the quality of the simulations of root canal preparations. In addition to constructing models to generate file geometry, we analyze the effect of the different geometric and kinematic manufacturing parameters on the final file geometry, thus highlighting the changes observed in the file cross-section. To the best of our knowledge, this is the first study in the scientific literature to simulate the generation of endodontic files produced via grinding manufacturing methods.

## 2. Description of the Endodontic File Manufacturing Process

**Figure 1:**
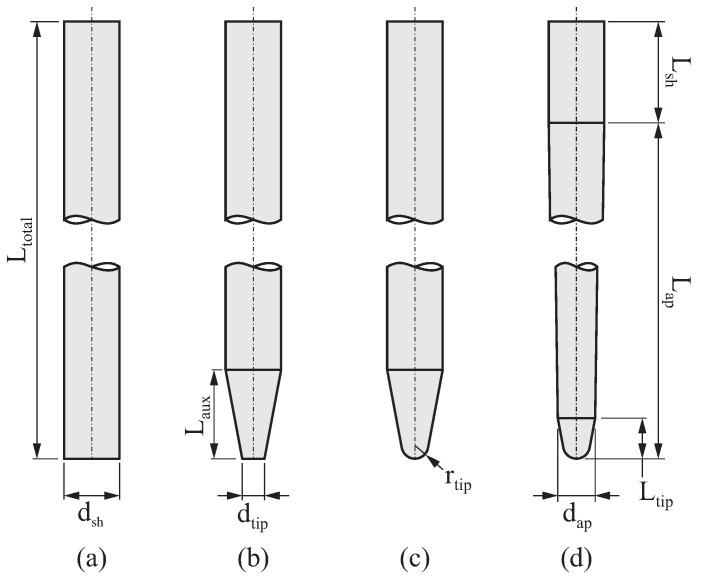
Creation of the blank of an endodontic file: main dimensions and shape. Geometries for (**a**) the raw material, (**b**) the initial shape of the tip, (**c**) the rounding of the tip and (**d**) the active part.

The manufacturing of endodontic files via a grinding process is described in U.S. patents 5.527.205 [24] and 5.807.106 [25]. Subsequently, several companies (such as *Royal Master* and *Rollomatic*) developed grinding machines to produce endodontic files with different dimensions and shapes. This manufacturing method is briefly summarized in three steps to provide the required background to understand the computational methods that are described in this paper:

**Step 1.** The raw material for the endodontic file is shaped into a cylinder with length $L_{total}$ and diameter $d_{sh}$ (Figure 1a).

**Step 2.** The raw material is shaped to create a blank for manufacturing the endodontic file:

1. Initially, the tip of the blank undergoes frustoconical grinding and is characterized by dimensions $d_{sh}$, $d_{tip}$, and $L_{aux}$ (Figure 1b).
2. Then, the tip of the blank is rounded with a radius $r_{tip}$ (Figure 1c). Radius $r_{tip}$ must be calculated from tangency conditions considering $d_{sh}$, $d_{tip}$, and $L_{aux}$ to maintain the dimensions of the blank and achieve a fully rounded tip.
3. Finally, the blank is subject to frustoconical grinding to shape the active part of the endodontic file (Figure 1d). The conicity of this part is characterized by dimensions $d_{sh}$, $L_{ap}$, and $d_{ap}$, where $d_{ap}$ is calculated from $L_{tip}$, $L_{aux}$, $d_{sh}$, and $d_{tip}$. The length of the shaft is defined as $L_{sh}=L_{total}-L_{ap}$.

**Figure 2:**
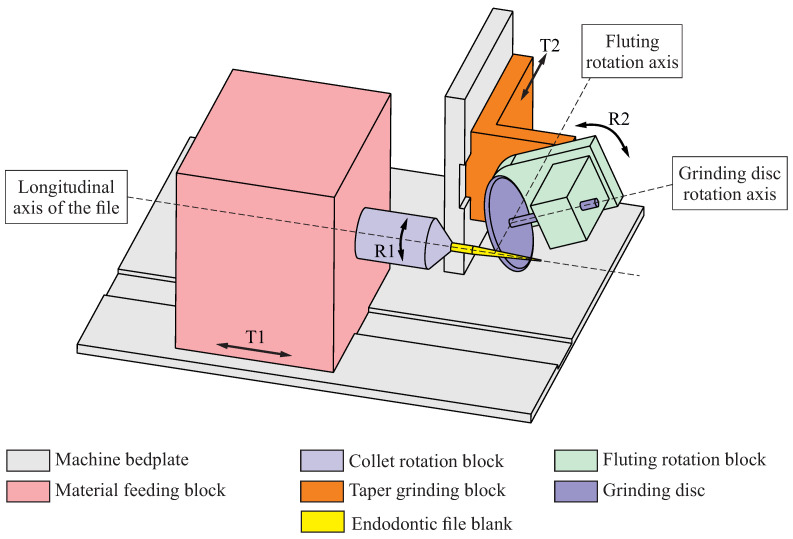
Schematic representation of the grinding process of the flutes of an endodontic file.

**Step 3.** A blank with the dimensions and shape of the final root canal instrument is obtained. Then, the helical flutes are machined onto this blank using a deep-grinding apparatus, which is akin to the one depicted in Figure 2. The basic setup for this machine is as follows:

1. The endodontic file blank is secured onto a collet that rotates around the longitudinal axis of the file, which is parallel to the machine bedplate (rotation movement R1 in Figure 2). The collet is attached to a feeding block that moves in the direction of the longitudinal axis of the file (translation movement T1 in Figure 2). The combination of movements R1 and T1 enables the blank to undergo both rotational and translational motions with respect to the machine bedplate.
2. The grinding wheel is supported by a fluting rotation block that can rotate around the fluting rotation axis, which is parallel to the machine bedplate and perpendicular to the longitudinal axis of the endodontic file (rotation movement R2 in Figure 2). The fluting rotation block is attached to a taper grinding block, which moves in the direction of the fluting rotation axis (translation movement T2 in Figure 2). The combination of movements R2 and T2 enables the grinding wheel to be positioned with respect to the endodontic file blank.
3. The grinding wheel rotates around an axis that is perpendicular to the fluting rotation axis. The machine is designed so that the geometrical center of the wheel coincides with the intersection of the grinding wheel and the fluting rotational axes.

The proper combination of the described movements (rotations R1 and R2, translations T1 and T2, and the rotation of the grinding wheel) allows the flute of the endodontic file to be machined. Repeating this combination of movements with an indexed initial position allows for the machining of several flutes using the endodontic file blank.

## 3. Mathematical Representation of the Geometry of an Endodontic File

In this section, we provide the mathematical expressions that define the geometry of the blank, the geometry of the flutes that are generated from the blank via the grinding process, and the geometry of the resulting endodontic file. Moreover, the mathematical expressions that define the lead $p_{z}$ and distance $\Delta_{xm}$ as a function of the generalized parameter of the flute grinding process ψ are provided.

### 3.1. Mathematical Representation of the Blank of an Endodontic File

**Figure 3:**
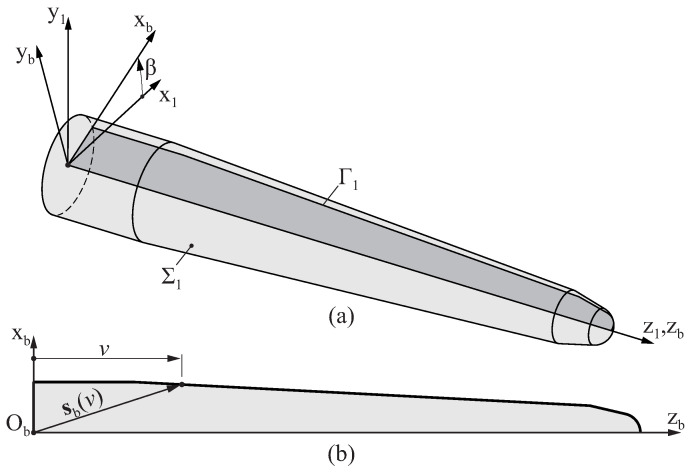
Schematic representation of the surface of the blank of an endodontic file, parameterized by the longitudinal coordinate *v* and the polar coordinate β: (**a**) Three-dimensional representation and (**b**) cross section of the blank. For clarity, the blank is not drawn to a realistic scale.

After the second step of the manufacturing process of the endodontic file described in Section 2, a blank is produced with the final dimensions of the endodontic file (see Figure 1d). The surface $\Sigma_{1}$ that defines the geometry of this blank is obtained by revolving a plane profile $\Gamma_{1}$ around axis $z_{1}$, as depicted in Figure 3a.

Figure 3b shows a generic profile of an endodontic file blank. The geometry of this profile is defined with respect to coordinate system $S_{b}$ using a parametric function $s_{b}\left(v\right)$ that provides the coordinates $\left(x_{b},z_{b}\right)$ of a point in the profile of the blank from its longitudinal intrinsic coordinate *v*.

Surface $\Sigma_{1}$ is represented in coordinate system $S_{1}$ as:

(1) $$s_{1}\left(v,\beta \right)=M_{1b}\left(\beta \right)\cdot s_{b}\left(v\right)$$

where β is the polar intrinsic coordinate of the blank surface, and $M_{1b}\left(\beta \right)$ is a homogeneous transformation matrix from coordinate system $S_{b}$ to coordinate system $S_{1}$ that is defined as

(2) $$M_{1b}\left(\beta \right)=\left[\begin{array}{cccc} \cos\beta & -\sin\beta & 0 & 0\\ \sin\beta & \cos\beta & 0 & 0\\ 0 & 0 & 1 & 0\\ 0 & 0 & 0 & 1 \end{array}\right]$$

### 3.2. Mathematical Representation of the Flutes of an Endodontic File

**Figure 4:**
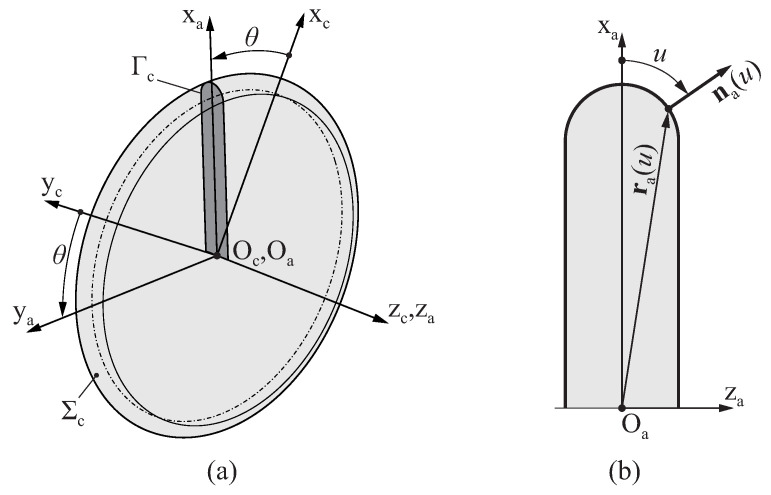
Schematic representation of the surface of a grinding wheel parametrized by the profile coordinate *u* and the polar coordinate θ: (**a**) Three-dimensional representation and (**b**) cross section of the grinding disk.

The surface $\Sigma_{c}$ that defines the geometry of the grinding wheel is determined by revolving a plane profile $\Gamma_{c}$ around axis $z_{c}$, as depicted in Figure 4a. Figure 4b shows a generic profile of a grinding wheel, which is defined with respect to coordinate system $S_{a}$ using a parametric function $r_{a}\left(u\right)$ that provides the coordinates $\left(x_{a},z_{a}\right)$ of a point in the profile of the grinding wheel from its profile intrinsic coordinate *u*. The Cartesian components of the unit vector that is normal to the wheel profile are given by parametric function $n_{a}\left(u\right)$.

Surface $\Sigma_{c}$ is represented in coordinate system $S_{c}$ as

(3) $$r_{c}\left(u,\theta \right)=M_{ca}\left(\theta \right)\cdot r_{a}\left(u\right)$$

where θ is the polar intrinsic coordinate of the grinding wheel surface, and $M_{ca}\left(\theta \right)$ is a homogeneous transformation matrix from coordinate system $S_{a}$ to coordinate system $S_{c}$, which is defined as follows:

(4) $$M_{ca}\left(\theta \right)=\left[\begin{array}{cccc} \cos\theta & -\sin\theta & 0 & 0\\ \sin\theta & \cos\theta & 0 & 0\\ 0 & 0 & 1 & 0\\ 0 & 0 & 0 & 1 \end{array}\right]$$

**Figure 5:**
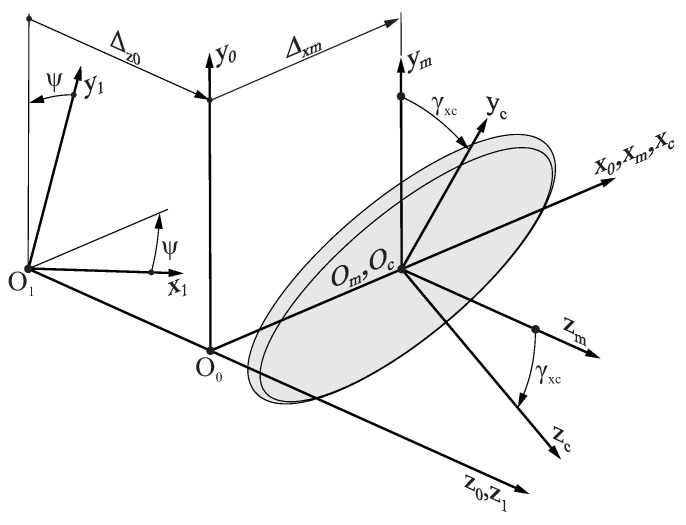
Coordinate systems used for the generation of the flutes of an endodontic file.

Figure 5 shows the coordinate systems that are used to represent the grinding wheel surface $\Sigma_{c}$ with respect to the different components of the grinding machine:

- Coordinate system $S_{0}$ is rigidly connected to the grinding machine bedplate, and its $z_{0}$ axis is aligned with the longitudinal axis of the endodontic file.
- Coordinate system $S_{m}$ is rigidly connected to the taper grinding block, which is parallel to coordinate system $S_{0}$, and its origin $O_{m}$ is located over the $x_{0}$ axis at a distance $\Delta_{xm}$ from $O_{0}$. The distance $\Delta_{xm}$ may vary during the grinding process to account for the taper of the endodontic file; thus, $\Delta_{xm}$ depends on the generalized parameter of the flute grinding process ψ.
- Coordinate system $S_{c}$ is rigidly connected to the grinding wheel. When assembled into the grinding machine setup, both the origin $O_{c}$ and the $x_{c}$ axis of $S_{c}$ are coincident with the origin $O_{m}$ and the $x_{c}$ axis of $S_{m}$. However, coordinate system $S_{c}$ is rotated by an angle $\gamma_{xc}$ around the $x_{m}$ axis in order to take into account the tilting of the grinding wheel with respect to the axis of the endodontic file. Angle $\gamma_{xc}$ is assumed to be constant throughout the entire flute generating process.
- Coordinate system $S_{1}$ is rigidly connected to the endodontic file, and its origin is located over the $z_{0}$ axis at a distance $\Delta_{z0}$ from $O_{0}$. Moreover, the $z_{0}$ and $z_{1}$ axes of these coordinate systems are coincident, but $S_{1}$ is rotated by an angle ψ with respect to $S_{0}$.

Coordinate transformation from $S_{c}$ to $S_{1}$ allows for the grinding wheel surface $\Sigma_{c}$ to be represented in its working position with respect to the endodontic file:

(5) $$r_{1}\left(u,\theta ,\psi \right)=M_{10}\left(\psi \right)\cdot M_{0m}\left(\psi \right)\cdot M_{mc}\cdot r_{c}\left(u,\theta \right)$$

where $M_{mc}$, $M_{0m}$, and $M_{10}$ are homogeneous transformation matrices defined as

(6) $$M_{mc}=\left[\begin{array}{cccc} 1 & 0 & 0 & 0\\ 0 & \cos\gamma_{xc} & -\sin\gamma_{xc} & 0\\ 0 & \sin\gamma_{xc} & \cos\gamma_{xc} & 0\\ 0 & 0 & 0 & 1 \end{array}\right]$$

(7) $$M_{0m}\left(\psi \right)=\left[\begin{array}{cccc} 1 & 0 & 0 & \Delta_{xm}\left(\psi \right)\\ 0 & 1 & 0 & 0\\ 0 & 0 & 1 & 0\\ 0 & 0 & 0 & 1 \end{array}\right]$$

(8) $$M_{10}\left(\psi \right)=\left[\begin{array}{cccc} \cos\psi & -\sin\psi & 0 & 0\\ \sin\psi & \cos\psi & 0 & 0\\ 0 & 0 & 1 & \Delta_{z0}\left(\psi \right)\\ 0 & 0 & 0 & 1 \end{array}\right]$$

Here, the $\Delta_{z0}\left(\psi \right)$ function relates the rotation of the blank to the translation of the material feeding block (movements T1 and R1 in Figure 2) through the lead $p_{z}$ of the file, which is defined as

(9) $$\Delta_{z0}\left(\psi \right)=\int_{0}^{\psi }p_{z}\left(\psi^{\prime }\right)\;d\psi^{\prime }$$

The geometry of the flute of an endodontic file is obtained as the envelope to the family of surfaces given by $r_{1}\left(u,\theta ,\psi \right)$, which is calculated by solving the following equation:

(10) $$\frac{\partial r_{1}}{\partial \psi }\cdot n_{1}=0$$

Here, $n_{1}$ and $\frac{\partial r_{1}}{\partial \psi }$ are defined as follows:

(11) $$n_{1}\left(u,\theta ,\psi \right)=M_{10}\left(\psi \right)\cdot M_{0m}\left(\psi \right)\cdot M_{mc}\cdot M_{ca}\left(\theta \right)\cdot n_{a}\left(u\right)$$

(12) $$\frac{\partial r_{1}}{\partial \psi }=\left(M_{10}\left(\psi \right)\cdot \frac{\partial M_{0m}\left(\psi \right)}{\partial \psi }+\frac{\partial M_{10}\left(\psi \right)}{\partial \psi }\cdot M_{0m}\left(\psi \right)\right)\cdot M_{mc}\cdot r_{c}\left(u,\theta \right)$$

where

(13) $$\frac{\partial M_{0m}\left(\psi \right)}{\partial \psi }=\left[\begin{array}{cccc} 1 & 0 & 0 & \frac{\partial \Delta_{xm}\left(\psi \right)}{\partial \psi }\\ 0 & 1 & 0 & 0\\ 0 & 0 & 1 & 0\\ 0 & 0 & 0 & 1 \end{array}\right]$$

(14) $$\frac{\partial M_{10}\left(\psi \right)}{\partial \psi }=\left[\begin{array}{cccc} -\sin\psi & -\cos\psi & 0 & 0\\ \cos\psi & -\sin\psi & 0 & 0\\ 0 & 0 & 1 & p_{z}\left(\psi \right)\\ 0 & 0 & 0 & 1 \end{array}\right]$$

### 3.3. Mathematical Representation of the Geometry of an Endodontic File

**Figure 6:**
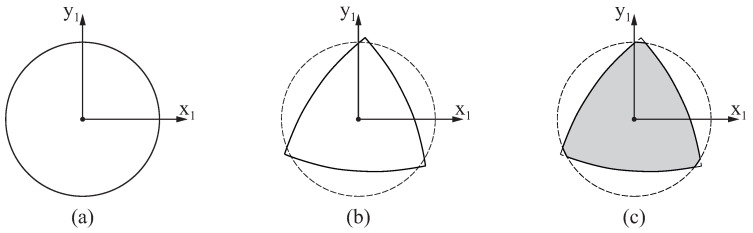
Cross-sections of the (**a**) blank, (**b**) flutes, and (**c**) endodontic file at an arbitrary longitudinal position.

Section 3.1 and Section 3.2 contain the mathematical expressions that define the geometry of the surface of the blank and the flutes of the endodontic file, respectively. The circular cross-section of the blank at any longitudinal position, defined by coordinate $z_{1}$, can be determined using Equation (1) and setting $v=z_{1}$ while varying β. Figure 6a shows an example of a cross-section of the blank.

The geometry of the flutes of an endodontic file can be determined by simultaneously considering Equations (5) and (10). The cross-section of the flutes at any longitudinal position defined by coordinate $z_{1}$ can be found by finding the values of θ and ψ that satisfy both Equation (10) and the following for each particular value of *u*:

(15) $$r_{1}\left(u,\theta ,\psi \right)\cdot k_{1}-z_{1}=0$$

where $k_{1}$ is the unit vector in the $z_{1}$-axis direction. Figure 6b shows an example of a cross-section of the flutes of an endodontic file with three flutes.

Overlapping both cross-sections, as illustrated in Figure 6c, allows us to define the geometry of the cross-section of the endodontic file at coordinate $z_{1}$. The geometry of the entire endodontic file can be determined by repeating these steps for different values of $z_{1}$.

### 3.4. Establishment of a Relation between $p_{z}$ and ψ

The application of Equation (9) to determine the magnitude of $\Delta_{z0}$ as a function of ψ requires of the definition of a function $p_{z}\left(\psi \right)$ that establishes a relation between the rotation and the translation of the blank during the flute generating process.

Even though $p_{z}\left(\psi \right)$ can take any shape, we assumed a linear relationship between the generation parameter ψ and the lead $p_{z}$ in this study, which can be mathematically expressed as

(16) $$p_{z}\left(\psi \right)=m_{p}\cdot \left(\psi -\psi_{i}\right)+b_{p}$$

where $m_{p}$ and $b_{p}$ are constants to be determined from the design parameters of the endodontic file, and $\psi_{i}$ corresponds to the value of ψ that satisfies $\Delta_{z0}\left(\psi_{i}\right)=L_{sh}$.

Considering Equation (9), the following explicit relationship between ψ and $\Delta_{z0}$ can be obtained:

(17) $$\Delta_{z0}\left(\psi \right)=\frac{m_{p}}{2}\cdot \psi^{2}+\left(b_{p}-\psi_{i}\cdot m_{p}\right)\cdot \psi$$

The following set of boundary conditions is considered to determine $m_{p}$, $b_{p}$, and $\psi_{i}$:

(18) $$\left\{\begin{array}{c} p_{z}\left(\psi_{i}\right)=p_{zi}\\ \Delta_{z0}\left(\psi_{i}\right)=L_{sh}\\ \Delta_{z0}\left(\psi_{f}\right)=L_{total}\\ p_{z}\left(\psi_{f}\right)=p_{zf} \end{array}\right.$$

where $p_{zi}$ is the lead at the beginning of the active part, $p_{zf}$ is the lead at the tip of the file, and $\psi_{f}$ corresponds to the value of ψ that satisfies $\Delta_{z0}\left(\psi_{f}\right)=L_{total}$. The following solutions for $b_{p}$, $m_{p}$, and $\psi_{i}$ are obtained with these boundary conditions:

(19) $$b_{p}=p_{zi}$$

(20) $$m_{p}=\frac{p_{zf}^{2}-p_{zi}^{2}}{2\cdot L_{ap}}$$

(21) $$\psi_{i}=\frac{p_{zi}-\sqrt{p_{zi}^{2}-2\cdot m_{p}\cdot L_{sh}}}{m_{p}}$$

The derivative of $\Delta_{z0}$ with respect to ψ, which may be used in further calculations, is

(22) $$\frac{\partial \Delta_{z0}\left(\psi \right)}{\partial \psi }=p_{zi}+\frac{p_{zf}^{2}-p_{zi}^{2}}{2\cdot L_{ap}}\cdot \left(\psi -\psi_{i}\right)$$

### 3.5. Establishment of a Relationship between $\Delta_{xm}$ and ψ

During the flute grinding process, the taper grinding block of the grinding machine moves toward the longitudinal axis of the file to account for the taper of the endodontic file. This movement is described by function $\Delta_{xm}\left(\psi \right)$, which provides the distance from the center of the grinding wheel to the longitudinal axis of the file as a function of ψ. Function $\Delta_{xm}\left(\psi \right)$ can take any arbitrary shape to obtain the geometry desired for the endodontic file.

**Figure 7:**
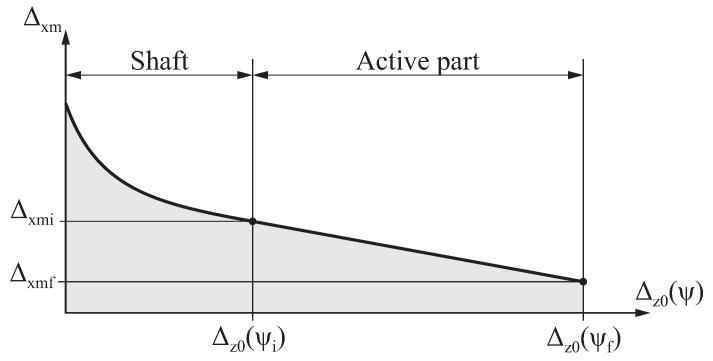
Definition of the approximation function $\Delta_{xm}\left(\psi \right)$.

Figure 7 shows the function $\Delta_{xm}\left(\psi \right)$ selected in this study, which is mathematically defined as follows:

(23) $$\Delta_{xm}\left(\psi \right)=\left\{\begin{array}{c} m_{a}\cdot \left[\Delta_{z0}\left(\psi \right)-L_{sh}\right]+d_{a}\;\mathrm{if}\;\psi \geq \psi_{i}\\ a_{a}\cdot {\left[\Delta_{z0}\left(\psi \right)-L_{sh}\right]}^{2}+b_{a}\cdot \left[\Delta_{z0}\left(\psi \right)-L_{sh}\right]+c_{a}\;\mathrm{if}\;\psi <\psi_{i} \end{array}\right.$$

where $a_{a}$ is a user-defined parameter; $m_{a}$, $d_{a}$, $b_{a}$, and $c_{a}$ are constants to be determined from the design parameters of the endodontic file. This function is linear in the active part of the file $\left(\psi \geq \psi_{i}\right)$ and parabolic in the shaft $\left(\psi <\psi_{i}\right)$, with the aim of quickly separating the grinding wheel from the file to avoid the excessive grinding of the shank.

The derivative of $\Delta_{xm}\left(\psi \right)$ with respect to ψ is calculated as

(24) $$\frac{\partial \Delta_{xm}\left(\psi \right)}{\partial \psi }=\frac{\partial \Delta_{z0}\left(\psi \right)}{\partial \psi }\cdot \left\{\begin{array}{c} m_{a}\;\mathrm{if}\;\psi \geq \psi_{i}\\ 2\cdot a_{a}\cdot \left[\Delta_{z0}\left(\psi \right)-L_{sh}\right]+b_{a}\;\mathrm{if}\;\psi <\psi_{i} \end{array}\right.$$

Assuming that $\Delta_{xm}\left(\psi_{i}\right)=\Delta_{xmi}$ and $\Delta_{xm}\left(\psi_{f}\right)=\Delta_{xmf}$, as well as by establishing $C^{1}$ continuity conditions at $\psi =\psi_{i}$, the following solutions for $m_{a}$, $d_{a}$, $b_{a}$, and $c_{a}$ are found:

(25) $$d_{a}=c_{a}=\Delta_{xmi}$$

(26) $$m_{a}=b_{a}=\frac{\Delta_{xmf}-\Delta_{xmi}}{L_{ap}}$$

## 4. Numerical Examples and Discussion

The performance of the proposed method in generating the geometry of endodontic files reproducing the actual flute grinding process is described in this section. Several case studies were considered for this purpose, with all of them having the same geometry for the initial blank of the endodontic file, which was defined by the parameters shown in Table 1 (corresponding to the blank shape and dimensions given in Figure 1).

Considering this initial blank, six representative endodontic file geometries were produced with different parameters for the flute grinding process, which are shown in Table 2.

**Figure 8:**
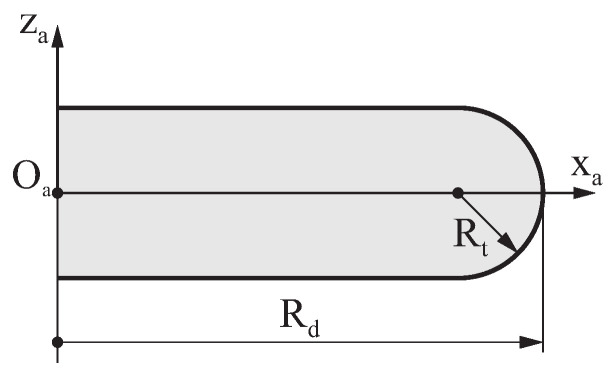
Parameterization of the geometry of the grinding wheel.

These parameters were classified into four categories:

- The geometry of the grinding wheel: The profile of the grinding wheel with rounded edges selected for the generation of the endodontic files is shown in Figure 8. This profile was parameterized by the radius of the grinding wheel $R_{d}$ and the tip radius $R_{t}$, which remained constant for all the case studies. Any other geometry could have been selected for the profile of the grinding wheel.
- The parameters that describe the movement of the material feeding block: A linear relationship between the generating parameter ψ and the lead $p_{z}$ was selected, which was parameterized by $p_{zi}$ and $p_{zf}$ (see Section 3.4). Case studies A to E included examples where the lead was constant throughout the entire length of the file, thus setting $p_{zi}=p_{zf}$. Case study F was an example of an endodontic file with a variable lead, where $p_{zi}>p_{zf}$.
- The parameters that describe the movement of the taper grinding block: As described in Section 3.5, in this study, a linear relationship between the generating parameter ψ and distance $\Delta_{xm}$ was selected for the active part, whereas a parabolic function was selected for the rest of the file. These functions were parameterized by $\Delta_{xmi}$, $\Delta_{xmf}$, and $a_{p}$. The values specified for these parameters were adjusted for each particular case.
- Other parameters of the grinding process included such factors as the tilt angle of the grinding wheel $\gamma_{xc}$ and the number of flutes *n*. All the case studies had three flutes, but any other integer value could be specified for this parameter. Different values were selected for the tilt angle of the grinding wheel $\gamma_{xc}$, which were obtained from $45^{\circ }$ variations over the magnitude of the reference helix angle $\alpha_{m}$ of the endodontic file (see Appendix A for further details).

Thus, following the method proposed in this study, the geometry of the flutes of the endodontic file was defined by a set of nine parameters: $R_{d}$, $R_{t}$, $p_{zi}$, $p_{zf}$, $\Delta_{xmi}$, $\Delta_{xmf}$, $a_{p}$, $\gamma_{xc}$, and *n* (the reference helix angle $\alpha_{m}$ is a derived parameter). Considering the six independent parameters that defined the geometry of the blank, the geometry of the endodontic file was fully defined by a set of 15 parameters.

**Figure 9:**
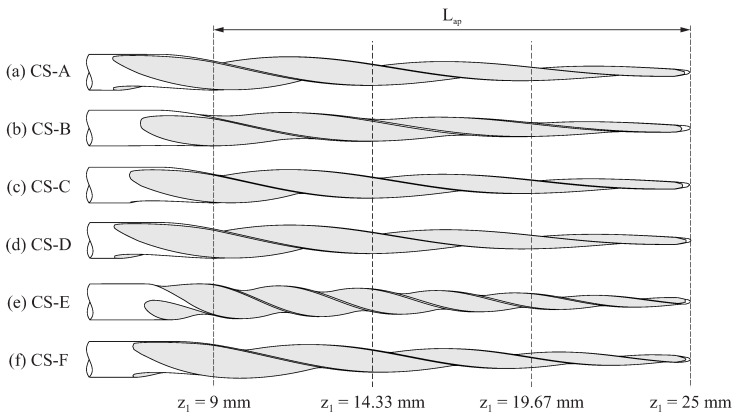
Lateral view for the case studies defined in Table 2.

**Figure 10:**
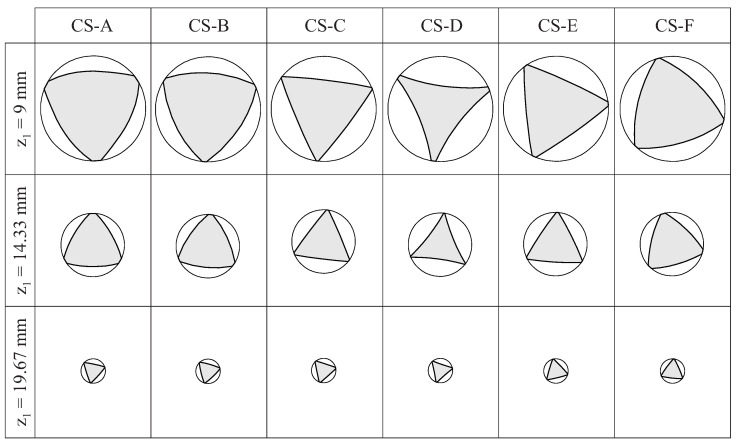
Cross-sections at different positions along the length of the endodontic files defined by the case studies defined in Table 2.

Figure 9 shows the lateral view for the selected case studies, and Figure 10 shows the cross-sections of these endodontic files at three different positions along the active part (which are defined in Figure 9). These figures evidence the effects of the grinding parameters on the geometry of the endodontic files.

Figure 10 shows that the shape of the cross-section changed along the length of the active part of the file. The differences between the cross-sections of the different files were more evident near the shank and became almost negligible near the file tip. These results indicate that an approximate file geometry, generated by simply rotating and scaling a section shape, as conducted in previous studies [15,20,32], is not realistic for this grinding manufacturing method.

Figure 10 shows that the flute grinding parameters affected the concavity/convexity of the file cross-section. A convex cross-section tends to be stronger and less elastic [33]; for this reason, narrow canals should be threaded during the initial phase of shaping. On the contrary, a concave cross-section tends to be more elastic but not as strong, so it may be more suitable for wider canals in the final phase of shaping. Thus, finding the specific combination of flute grinding parameters to obtain optimum curvatures for the cross-section of the files may result in better clinical outcomes.

We next investigated the effect of the tilt angle of the grinding wheel $\gamma_{xc}$, the lead $p_{z}$, the distance $\Delta_{xm}$, and the tip radius of the grinding wheel $R_{t}$ on the geometry of the endodontic file.

### 4.1. Effect of the Tilt Angle of the Grinding Wheel

Case studies A, B, C, and D were used to demonstrate the effect of changing the tilt angle of the grinding wheel ($\gamma_{xc}$) while keeping the lead constant and uniform along the file length. A change in $\gamma_{xc}$ modifies the relative position of the grinding wheel with respect to the longitudinal axis of the endodontic file, thus affecting how the material is removed from the blank during the flute grinding process.

**Figure 11:**
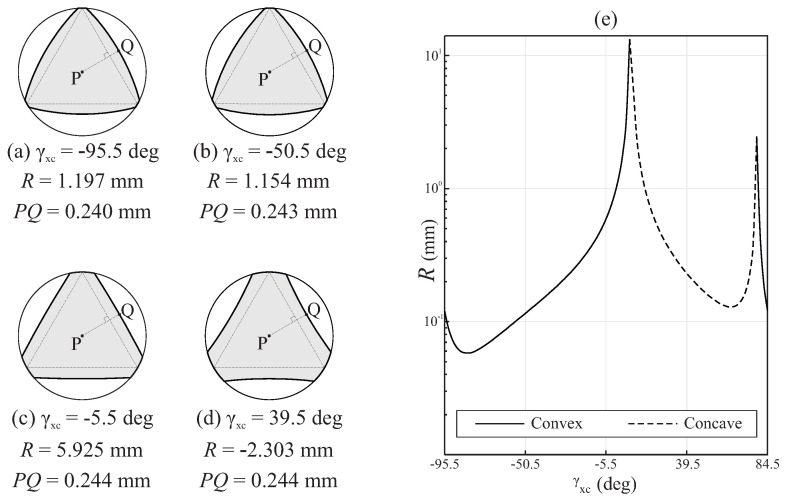
Analysis of the influence of $\gamma_{xc}$ on the geometry in case study B: (**a**–**d**) geometry of the cross-section at the middle of the active part for different values of $\gamma_{xc}$ and (**e**) evolution of the radius of the curvature of the cross-section with $\gamma_{xc}$.

Figure 11a–d show the cross-section at the middle of the active part for different variations of case study B, where $\gamma_{xc}$ was changed, while the rest of the design parameters remained constant, to demonstrate the independent effects of $\gamma_{xc}$ over the geometry of the file. The dashed line draws an equilateral triangle circumscribed within the section of the file. This triangle has been properly aligned with the cross-section of the file to facilitate the visualization of the differences in the cross-section among the cases. Moreover, the figure includes a dashed line PQ that is perpendicular to the edge of this triangle. Point *P* indicates the center of the cross-section, and point *Q* indicates the intersection of this line with the edge of the cross-section.

In all the cases shown in Figure 11a–d, each edge of the cross-section of the file corresponds to a segment of a circumference, with a radius denoted by *R*, as indicated in Figure 11a–d, together with distance PQ. Regardless of the value of $\gamma_{xc}$, point *Q* always corresponds to the point on the edge of the cross-section that was either at a maximum or minimum distance of *P* (for convex and concave cross-sections, respectively). The results show that the effect of $\gamma_{xc}$ over distance PQ was negligible in this case study.

Figure 11e shows the variation in the radius of the curvature of the edges of the cross-section at the middle of the active part of case study B when only $\gamma_{xc}$ was changed (note that cases with $\gamma_{xc}=-95.5{}^{\circ}$ and $\gamma_{xc}=84.5{}^{\circ}$ were equivalent). Here, depending on the value of $\gamma_{xc}$, a convex or concave cross-section was obtained. The change from a convex to a concave cross-section (and vice versa) is characterized by a singularity point, where *R* tends to infinity, thus leading to a cross-section with flat edges. In this particular case, these singularity points were observed when $\gamma_{xc}≃14.5{}^{\circ}$ and $\gamma_{xc}≃83.5{}^{\circ}$. In the interval $14.5{}^{\circ}<\gamma_{xc}<83.5{}^{\circ}$, the cross-section was concave (Figure 11d), whereas in the rest of the domain, the cross-section was convex (Figure 11a–c).

### 4.2. Effect of the Lead

The lead defines the relationship between the translation and the rotation of the file during the flute grinding process; in this study, the lead was characterized by parameters $p_{zi}$ and $p_{zf}$ (Section 3.4). A constant lead throughout the length of the file was achieved by setting $p_{zi}=p_{zf}$, and two examples of endodontic files with a constant lead are shown in Figure 9c (case study C) and Figure 9e (case study E). The proposed approach also allowed us to generate flute geometries where the lead linearly varied from the beginning to the end of the active part, as shown in Figure 9f for case study F.

The lead of the manufacturing process affects the pitch of the file, which has clinical implications. Increasing the lead (which increases the pitch of the files) reduces the tendency to screw in and the torsional load sharing [34]. Decreasing the pitch (which reduces the pitch of the files) increases the cutting efficiency, the bending stiffness, and the fatigue life of a file [32,35]. Finally, the use of variable lead files helps to reduce the amount of cutting debris retained in the flutes [36].

**Figure 12:**
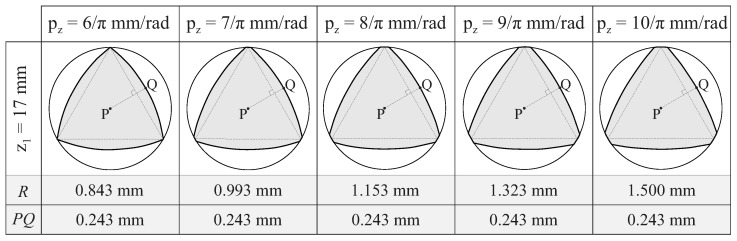
Cross-section at the middle of the active part for different variants of case study B, where a constant lead $p_{z}=p_{zi}=p_{zf}$ was varied, while all other design parameters remained constant.

Figure 12 shows different variants of case study B, where $p_{zi}=p_{zf}$ was changed, while the rest of the design parameters remained constant, to demonstrate the independent effects of the lead on the geometry of file’s cross-section.

In all the cases shown in Figure 12, each edge of the cross-section of the file corresponds to a segment of a circumference with a radius denoted by *R*, as indicated in Figure 12, together with distance PQ. Regardless of the value of $p_{zi}=p_{zf}$, point *Q* always corresponds to the point on the edge of the cross-section that is at a maximum distance from *P*, and the distance PQ was not affected by variations in the lead. Decreasing and increasing the lead of the file tended to decrease and increase the curvature radius of the edges of the cross-section, respectively.

According to Equation (A3), by keeping the rest of the parameters constant, the reference helix angle of the file decreased as the magnitude of the lead increased. In the cases shown in Figure 12, the reference helix angle $\alpha_{m}$ changed from −7.3 ° (when $p_{z}=6\;\mathrm{mm}/\mathrm{rad}$) to −4.4 ° (when $p_{z}=10\;\mathrm{mm}/\mathrm{rad}$).

### 4.3. Effect of the Approximation at the Beginning of the Active Part

**Figure 13:**
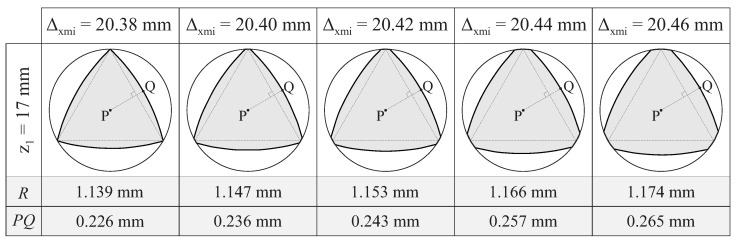
Cross-section at the middle of the active part for different variants of case study B, where the approximation at the beginning of the active part $\Delta_{xmi}$ was varied, while all other design parameters remained constant.

As the flute is being generated, the taper grinding block approaches the longitudinal axis of the file, thus following a function $\Delta_{xm}\left(\psi \right)$ that was characterized by $\Delta_{xmi}$, $\Delta_{xmf}$ and $a_{p}$ in this study (Section 3.5). Figure 13 shows different variants of case study B, where $\Delta_{xmi}$ was changed, while the rest of parameters remained constant, to demonstrate the independent effects of these parameters on the geometry of the endodontic file.

In all the cases shown in Figure 13, each edge of the cross-section of the file corresponds to a segment of a circumference with a radius denoted by *R*, as indicated in Figure 13, together with distance PQ. Regardless of the value of $\Delta_{xmi}$, point *Q* always corresponds to the point on the edge of the cross-section that is at the maximum distance from *P*.

The results obtained from this analysis revealed that increasing $\Delta_{xmi}$ tended to increase the curvature radius of the edges of the cross-section, even though this increase was almost negligible. The distance PQ increased as $\Delta_{xmi}$ increased, thus also increasing the area of the cross-section.

When $\Delta_{xmi}$ was changed while $\Delta_{xmf}$ was kept constant, the slope of the approximation function changed. As such, in the examples shown in Figure 13, the slope changed from 1.9% (when $\Delta_{xmi}=20.38\;\mathrm{mm}$) to 2.4% (when $\Delta_{xmi}=20.46\;\mathrm{mm}$). These slopes can be compared with the slope of the file blank, which was 3% in all the case studies.

According to Equation (A3), the helix angle of the file increases with the magnitude of the approximation function. In the cases shown in Figure 13, the reference helix angle $\alpha_{m}$ changed from −5.1 ° (when $\Delta_{xmi}=20.38\;\mathrm{mm}$) to −6.0 ° (when $\Delta_{xmi}=20.46\;\mathrm{mm}$).

### 4.4. Effect of the Tip Radius of the Grinding Wheel

**Figure 14:**
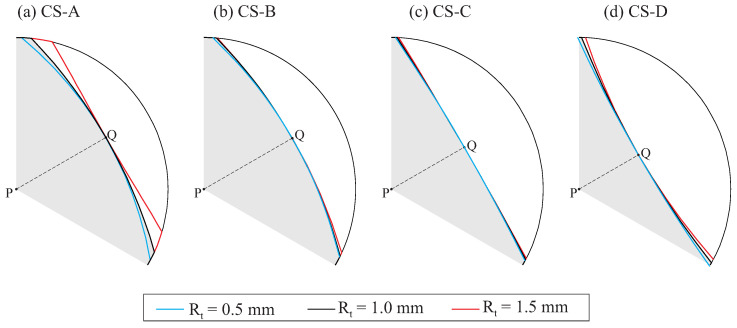
Partial view of the flute cross-section in the middle of the active part for case studies A, B, C, and D for variations in $R_{t}$.

Finally, the effect of the tip radius $R_{t}$ on the geometry of the endodontic file was investigated. Figure 14 illustrates the effect of differences in the tip radius of the grinding wheel ($R_{t}$ in Figure 8) on the geometry of the endodontic files for case studies A to D, when the rest of the design parameters remained constant. For clarity, only one-third of the resulting cross-section in the middle of the active part is shown in this figure.

Figure 14 shows that varying $R_{t}$ changed the curvature of the edges of the cross-section of the file, thus increasing their radius of curvature as $R_{t}$ increased. The distance PQ did not change with $R_{t}$; thus, the area of the cross-section increased as $R_{t}$ increased.

## 5. Conclusions

We developed a method for generating the geometry of endodontic files by reproducing the flute grinding process. This approach provides realistic file geometries, including files with a variable lead, using fifteen independent parameters that define the file manufacturing process.

The performance of the method was demonstrated through several case studies, which led to the following conclusions:

- The tilt angle of the grinding wheel $\gamma_{xm}$ affects the curvature of the edges of the cross-section of the file. An appropriate selection of $\gamma_{xm}$ can lead to convex and concave cross-section geometries, as well as cross-sections with straight edges. The effects of $\gamma_{xm}$ on the distance PQ are negligible.
- The lead of the file, characterized by $p_{zi}$ and $p_{zf}$, affects the helix angle of an endodontic file and the curvature of its cross-section. Decreasing the lead of the file decreases the curvature radius of the edges of its cross-section. The distance PQ is not affected by changes in the lead.
- The distance between the grinding wheel and the axis of rotation of the file is characterized by $\Delta_{xmi}$, $\Delta_{xmf}$, and $a_{p}$. Changing $\Delta_{xmi}$ does not affect the curvature of the edges of the cross-section but strongly influences distance PQ.
- The tip radius of the grinding wheel $R_{t}$ has a different effect on the geometry of the endodontic file depending on the settings of the grinding machine. In general, the radius of curvature of the edges of the cross-section increases with $R_{t}$.

The computational method developed in this study is a valuable tool for designing and manufacturing root canal files with specific geometric features in a controlled manner. To the best of our knowledge, this is the first study in the scientific literature to simulate the generation of endodontic files by reproducing grinding manufacturing methods.

The scientific literature shows that the geometric design of an endodontic file determines its mechanical properties, i.e., service life, cutting efficiency, flexibility, and tendency to ledge or screw in, which has important clinical implications. For this reason, the realistic virtual geometries obtained with the proposed method will help to increase the quality of FE simulations used to understand the mechanical response of endodontic files in clinical service, thereby contributing to reducing the failure rates and improving clinical practices in endodontic therapy.

Future work could address a more systematic analysis of the different manufacturing parameters on the final generated geometry. The recommended ranges for these parameters and their combinations to obtain valid geometries should also be analyzed.

## Figures and Tables

**Table 1:** Design parameters of the blank of the endodontic file according to Figure 1. Parameters $L_{sh}$, $r_{tip}$, $d_{ap}$, and *c* were calculated from the other parameters.

Parameter	Magnitude
Shaft diameter: $d_{sh}$	1.2mm
Total length: $L_{total}$	25.0mm
Tip diameter: $d_{tip}$	0.096mm
Auxiliary tip length: $L_{aux}$	3.0mm
Length of active part: $L_{ap}$	16.0mm
Tip length: $L_{tip}$	0.5mm
Shaft length: $L_{sh}$	9.0mm
Rounding radius of the tip: $r_{tip}$	0.057mm
Diameter of active part transition: $d_{ap}$	0.27mm
Taper of active part: *c*	6%

**Table 2:** Cases in this study defined by the parameters of the grinding process.

Case Study	A	B	C	D	E	F
Grinding wheel radius: $R_{d}$ (mm)	20	20	20	20	20	20
Tip radius: $R_{t}$ (mm)	1.0	1.0	1.0	1.0	1.0	1.0
Initial lead: $p_{zi}$ (mm/rad)	8/π	8/π	8/π	8/π	4/π	8/π
Final lead: $p_{zf}$ (mm/rad)	8/π	8/π	8/π	8/π	4/π	4/π
Initial distance: $\Delta_{xmi}$ (mm)	20.42	20.42	20.33	20.26	20.42	20.34
Final distance: $\Delta_{xmf}$ (mm)	20.07	20.07	20.07	20.07	20.07	20.07
Parabola coefficient: $a_{p}$ (mm^−1^)	0.03	0.03	0.03	0.03	0.03	0.03
Reference helix angle: $\alpha_{m}$ (deg)	−5.6	−5.5	−4.5	−3.7	−10.5	−5.5
Grinding wheel angle: $\gamma_{xc}$ (deg)	−95.6	−50.5	−4.5	40.5	−10.5	−5.5
Number of flutes: *n*	3	3	3	3	3	3

**Figure A1:**
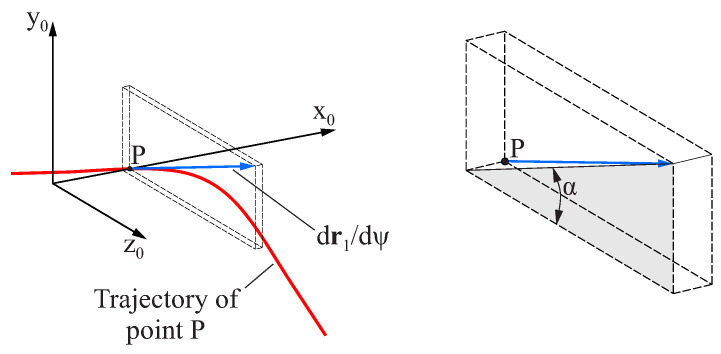
Definition of the helix angle α(ψ).

## Data Availability

The dataset is available upon request from the authors.

## Appendix A. Calculation of the Reference Helix Angle of the Endodontic File

Figure A1 shows the trajectory of point *P* of the grinding wheel surface, which is placed over the $x_{c}$ axis of coordinate system $S_{c}$ (which is coincident with axis $x_{0}$ of coordinate system $S_{0}$) during the flute generating process. The position of point *P* in coordinate system $S_{c}$ is given by

(A1) $$r_{c}\left(0\;\mathrm{mm},\pi \;\mathrm{rad}\right)={\left[\begin{array}{cccc} -R_{d} & 0 & 0 & 1 \end{array}\right]}^{T}$$

Here, $R_{d}$ is the external radius of the grinding wheel. The derivative of $r_{1}\left(0,\pi ,\psi \right)$ with respect to ψ is a vector that is tangent to the trajectory described by that point during the generating process, which can be calculated using Equation (12). Coordinate transformation from $S_{1}$ to $S_{0}$ describes such a vector in coordinate system $S_{0}$ as

(A2) $$\frac{\partial r_{1}\left(\psi \right)}{\partial \psi }{|}_{S_{0}}={\left[\begin{array}{cccc} \frac{\partial \Delta_{xm}\left(\psi \right)}{\partial \psi } & \Delta_{xm}\left(\psi \right)-R_{d} & p_{z}\left(\psi \right) & 0 \end{array}\right]}^{T}$$

The helix angle α, which is defined as the angle between $z_{0}$ and the projection of the previous vector onto the $y_{c}z_{c}$ plane, can be calculated as

(A3) $$\alpha \left(\psi \right)=\arctan\left(\frac{\Delta_{xm}\left(\psi \right)-R_{d}}{p_{z}\left(\psi \right)}\right)$$

This equation shows that the helix angle depends on $\Delta_{xm}$ and $p_{z}$, whose values change with ψ throughout the length of the file, thus implying that the helix angle also changes throughout the length of the file. The reference helix angle $\alpha_{m}$ is defined as follows to simplify further calculations:

(A4) $$\alpha_{m}=\alpha \left(\frac{\psi_{i}+\psi_{f}}{2}\right)$$
